# SLy2‐deficiency promotes B‐1 cell immunity and triggers enhanced production of IgM and IgG_2_ antibodies against pneumococcal vaccine

**DOI:** 10.1002/iid3.365

**Published:** 2020-10-24

**Authors:** Jennifer Jaufmann, Leyla Tümen, Fee Schmitt, Daniel Schäll, Max von Holleben, Sandra Beer‐Hammer

**Affiliations:** ^1^ Department of Pharmacology, Experimental Therapy and Toxicology, Institute of Experimental and Clinical Pharmacology and Pharmacogenomik and ICePhA University of Tuebingen Tuebingen Germany; ^2^ Institute for Medical Microbiology and Hospital Hygiene Heinrich‐Heine‐University Duesseldorf Germany

**Keywords:** antibody responses, B‐1 cells, natural IgM, pneumococcal vaccination, pneumonia, Src homology domain 3 lymphocyte protein 2, *Streptococcus pneumoniae*

## Abstract

**Background:**

Despite the benefits of existing vaccines, *Streptococcus pneumoniae* is still responsible for the greatest proportion of respiratory tract infections around the globe, thereby substantially contributing to morbidity and mortality in humans. B‐1 cells are key players of bacterial clearance during pneumococcal infection and even provide long‐lasting immunity towards *S. pneumoniae*. Previous reports strongly suggest an essential role of the immunoinhibitory adapter Src homology domain 3 lymphocyte protein 2 (SLy2) for B‐1 cell‐mediated antibody production. The objective of this study is to evaluate *S. pneumoniae*‐directed B cell responses in the context of SLy2 deficiency.

**Methods:**

B‐1 cell populations were analyzed via flow cytometry before and after pneumococcal immunization of SLy2‐deficient and wild‐type control mice. Global and vaccine‐specific immunoglobulin M (IgM) and IgG antibody titers were assessed by enzyme‐linked immunosorbent assay. To investigate survival rates during acute pneumococcal lung infection, mice were intranasally challenged with *S. pneumoniae* (serotype 3). Complementary isolated splenic B cells were stimulated in vitro and their proliferative response was assessed by fluorescent staining. In vitro antibody secretion was quantified by LEGENDplex.

**Results:**

We demonstrate increased frequencies of B‐1 cells and elevated titers of preantigenic IgM in SLy2‐deficient mice. In addition, these mice produce significantly more amounts of IgM and IgG_2_ upon pneumococcal vaccination. Knocking out SLy2 did not induce survival advantages in our murine model of acute pneumonia, indicating the presence of compensatory mechanisms.

**Conclusion:**

Our results reveal reinforced specific antibody responses towards pneumococcal polysaccharides and enhanced IgG_2_ secretion as a consequence of SLy2 deficiency, which could be relevant to the development of more efficient vaccines.

## Graphical abstract

We previously defined the adapter protein SLy2 as an inhibitor of B‐1 cell‐mediated antibody production. To investigate immune cell responses in the absence of SLy2 in the context of pneumococcal immunization, we have generated SLy‐knockout (Ko) mice. The current study reports enriched B‐1 cell populations and natural IgM in SLy2‐Ko mice. Furthermore, a SLy2 deficiency significantly improved pneumococcal polysaccharide‐specific IgM and IgG2 responses upon vaccination. This study provides important insights into B cell immunity against *Streptococcus pneumoniae* and identifies the adapter protein SLy2 as a potential future therapeutic target in the course of vaccine optimization and development.

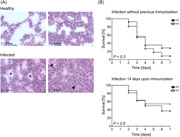

**Abbreviations**
BM: bone marrow
BMSC: bone marrow stromal cells
DS: Down syndrome
Ig: immunoglobulin
IPD: invasive pneumococcal disease
Ko: knockout
LPS: lipopolysaccharide
P23: Pneumovax 23
PCV13: Prevenar 13
pPS: pneumococcal polysaccharide
SLy2: Src homology domain 3 lymphocyte protein 2
TD: thymus/T cell‐dependent
Tg: transgenic
TI: thymus/T cell‐independent
TLR: Toll‐like receptor

## 1 INTRODUCTION

Src homology domain 3 lymphocyte protein 2 (SLy2) is an immunoinhibitory adapter that is encoded on human chromosome 21 and belongs to a group of three highly homologous protein family members (SLy1, SLy2, and SASH1). It is expressed broadly throughout several tissues including heart, muscles, brain, and the hematopoietic system.^1^, ^2^ Differential expression of SLy2 has been brought into context with a variety of human diseases such as solid tumors, multiple myeloma, and Down syndrome (DS).^3^, ^4^, ^5^, ^6^

SLy2 is localized in both the cytoplasm and the nucleus, suggesting nucleocytoplasmic shuttling of the protein. This hypothesis is further supported by the fact that it contains a bipartite nuclear‐localization signal at its N‐terminal end.^7^ In addition, SLy2 holds an Src homology 3 and a sterile alpha motif domain, both of which are crucial to its function. As a classical adapter protein, SLy2 mediates the formation and localization of protein complexes, thereby contributing to the transmission of intracellular signaling cascades. It has been reported to colocalize with huge complexes that control gene transcription and was shown to be involved in the regulation of actin dynamics and cell spreading.^7^, ^8^, ^9^

In immunological terms, studies collectively point to a role of SLy2 as an inhibitor of B‐1 cell activation and function. SLy2‐overexpressing transgenic (Tg) mice display normal T‐cell development, regular numbers of monocytes, dendritic cells, and granulocytes. However, these mice hold a significantly lower proportion of B‐1 cells, accompanied by reduced levels of natural serum IgM. Moreover, SLy2 overexpression attenuates interleukin (IL) 5‐dependent antibody production of stimulated B‐1 cells in vitro.^10^

B‐1 cells constitute an innate‐like B‐cell population, phenotypically and functionally differing from conventional (B‐2) B cells. They are mainly localized in the peritoneal and pleural cavity, possess the ability of self‐renewal, and display a limited repertoire of B‐cell receptor specificities. B‐1 cells constitutively secret natural IgM, targeting autoantigens on apoptotic, potentially damaging cells and toxic metabolites. In addition, they are key players of innate immunity, since they act as the main defense against numerous bacterial pathogens. Upon antigen recognition, B‐1 cells rapidly respond in a T‐cell independent (TI) manner and differentiate into short‐lived plasma cells. In mice, B‐1 cells are clearly defined as CD19^+^CD43^+^IgM^+^CD23^−^ and can be subdivided into B‐1a and B‐1b cells, being CD5^+^ and CD5^−^, respectively.^11^, ^12^ The phenotype of human B‐1 cells is still controversially discussed; however, there exists a B‐1 cell‐like subset in humans sharing the functional and phenotypical characteristics of murine B‐1 cells.^13^

B‐1 cells substantially contribute to the clearance of pneumococcal antigens and even confer long‐lasting immunity against *Streptococcus pneumoniae*.^14^, ^15^ *S. pneumoniae* (Pneumococcus) is a commensal of the upper respiratory tract in humans, asymptomatically carried by a majority of the population. Upon stable colonization and immune evasion, *S. pneumoniae* can cause severe infectious diseases associated with high mortality rates such as pneumonia, otitis media, and sepsis. The risk of developing the invasive pneumococcal disease (IPD) is especially given in infants, the elderly and immune‐deficient subjects such as patients with DS.^16^, ^17^ DS goes ahead with significantly reduced levels of serum IgM and high susceptibility towards infections. A Swedish study identified infectious pneumonia as a leading cause of death in patients with DS.^18^ Intriguingly, SLy2 is amongst a small group of nine genes additionally amplified in DS.^3^ This led us to hypothesize that the overexpression of SLy2 in patients with DS may contribute to their increased susceptibility towards pneumococcal infection, by suppressing B‐1 cell responses directed against *S. pneumoniae*. Indeed, when immunized with the pneumococcal vaccine, SLy2‐Tg mice show significantly impaired antibody responses towards pneumococcal polysaccharides (pPS).^10^

Routine vaccinations of adults with the polysaccharide vaccine Pneumovax 23 (P23) and children with the conjugate‐vaccine Prevenar 13 (PCV13) promote herd immunity against *S. pneumoniae* by reducing nasopharyngeal colonization within the population. However, immune responses to pneumococcal vaccine decline with increasing age, and the overall efficacy of immunization in preventing adult pneumococcal lung infection is estimated to be weak.^19^, ^20^, ^21^ Hence, it is important to intensify our understanding of underlying innate and adaptive immune responses towards pneumococcal antigens as a pre‐requisite for the development of advanced vaccines.

To investigate whether B cell responses towards pneumococcal antigens are improved in the absence of SLy2, we generated a SLy2‐knockout (Ko) mouse model. We analyzed B‐1 cell populations, performed B‐cell stimulation experiments in vitro, and measured antibody levels of these mice before and after immunization with pneumococcal vaccine. Complementary, mice were challenged with *S. pneumoniae* to evaluate their survival in the course of acute lung infection.

Our data reveal increased frequencies of bone‐marrow (BM) resident B‐1b cells in SLy2‐Ko mice, accompanied by elevated levels of preantigenic immunoglobulin (Ig) M. In addition, Ko mice produced significantly more amounts of specific IgM and IgG_2_ antibodies upon vaccination, demonstrating an inhibitory role of SLy2 in the response towards P23 and PCV13. Surprisingly, the loss of SLy2 did not improve the survival rate of mice during acute lung infection with *S. pneumoniae* serotype 3, possibly due to the superiority of antibody‐independent mechanisms in our model.

## 2 MATERIALS AND METHODS

### 2.1 Generation, breeding, and maintenance of SLy2‐Ko mice

For the generation of SLy2‐Ko mice, we applied a fosmid derived cloning strategy, which makes use of fosmid vectors consisting of a long stretch of genomic DNA (∼40–60 kb) cloned into a plasmid, allowing for propagation in *Escherichia coli* in the presence of chloramphenicole. To generate the targeting construct, a *neoR* cassette was inserted into the target site via homologous recombination (Red/ET cloning system; Gene Bridges). The first step of the cloning reaction was the electroporation of the fosmid‐carrying *E. coli* with an inducible bacterial expression plasmid (pBAD) for the λ‐ recombinases Redα and Redβ that contains a temperature‐sensitive origin of replication (*oriR101*), allowing the propagation of the plasmid only up to 30°C. This rendered the bacteria chloramphenicole/tetracycline double resistant. These double resistant clones were picked, grown, and expression of the recombinase was induced by the addition of 1 M l‐arabinose solution and incubation of the cells at 37°C. Subsequently, 200 ng of the *neoR*‐cassette containing short arms (50 bp on either side) homologous to the site of insertion were electroporated into the bacteria (primer sequence forward: CGCAGCAGCAGTTTTGGGAATTTTGACCGTTTTCGGAATAATTCCGTATCGCCTTAACGTTGGAAAAGCTG; reverse: CTCTTCTCCTGCTTCTGGGGACCTTTATCTTCTTAGGAGCTGCTTCCTCTTCCG ATCGCCTAGGGGTAACC). As the *neoR*‐cassette contains a kanamycine resistance gene, bacteria harboring a successful recombination event were double‐resistant against chloramphenicole and kanamycine. Double resistant clones were picked, and recombination was assessed by polymerase chain reaction (PCR), restriction digest, and finally by southern blot. The *HSV‐TK* cassette was inserted 3′ of the homology region with the same method, using an expression cassette with an additional ampicilline resistance gene (primer sequence forward: GGATCCCCGGGTACCGAGCTCGAATTCGCCCTATAGTGAGTCG TATTACAATCGAGCAGTGTGGTTTTGC; reverse: GGTAACGCCAGGGTTTTCCCAGTCACGACGTT GTAAAACG ACGGCCAGTGAAGGTCATGAGATTATCAAAAAGG).

E14.1 embryonal stem (ES) cells were transfected by electroporation with the SLy2 targeting construct. To this end, 5 × 10^7^ cells were mixed with 200 μg of the linearized targeting construct in 1 ml phosphate‐buffered saline (PBS). Eight hundred microliters of this mix were electroporated in a Biorad Gene pulser II at 340 V and 250 μF. After 10 min of incubation on ice, cells were resuspended in prewarmed ES medium and the content of each cuvette was plated on two 10‐cm dishes with feeder cells. Selection of recombinant ES cells after electroporation was performed by supplementation of 200 μg/ml neomycine and 2 μM ganciclovir, favoring cells that had incorporated the targeting construct in a homologous recombined fashion, without incorporation of the HSV‐TK gene. C57BL/6 donor blastocysts were injected each with homologous recombined ES cells and blastocysts were implanted into CD1 foster mothers in a state of mock pregnancy. Chimeric mice were mated to C57BL/6 animals and the resulting offspring was analyzed for germline transmission of the mutation by southern blot and PCR.

All mice were kept and bred under specific pathogen‐free conditions in open cages. The SLy2‐Ko line was backcrossed to its C57BL6/N background for at least six generations. For immunization studies, 9–13‐week‐old female or male age‐matched littermates were used. For a ranasal challenge with *S. pneumoniae*, 16–17‐week old mice were utilized. All animal work was performed according to the German animal care regulations and animal experiments were approved by the local ethics committee (AZ G58/06; AZ 29.03.2017; PH5/11; PH1/14; and PH2/19).

### 2.2 Immunization and preparation protocols

To study cellular and humoral immune responses towards a pneumococcal vaccine, SLy2‐wild‐type (Wt) and Ko littermates were immunized with either 1 µg P23 (SanofiPasteurMSD) or 3 µg PCV13 (Pfizer) in 100 µl PBS intraperitoneally (ip). Complementarily, mice were immunized with 2 µg trinitrophenyl hapten (TNP)–lipopolysaccharide (LPS) in 200 µl PBS. For P23 and LPS, blood was collected before and 4, 7, 14, and 21 days after immunization (retrobulbar blood sampling). For PCV13 studies, mice were killed before and 7, 14, and 21 days after immunization to collect blood, peritoneal cells, spleen, and BM.

All blood samples were collected in Microtainer® blood collection tubes (BD Bioscience). After at least 30 min of incubation at room temperature (RT), tubes were centrifuged at 15,000*g* for 90 s to collect the serum in the supernatant. Sera were stored at −20°C. Peritoneal lavage (PL) was performed with 5 ml of ice‐cold PBS. Femurs were flushed out with 5 ml of ice‐cold PBS to harvest BM cells. After one washing step, PL and BM cells were directly used for further analysis. Spleens were homogenized using a 70 µm cell strainer and subsequently incubated with erythrocyte lysis buffer to get rid of red blood cells before analysis.

### 2.3 Infection experiments

For the challenge of mice with *S. pneumoniae* (ATCC strain 6303, serotype 3), an inoculated ring from a Roti®‐Store Cryo tube was transferred from −80°C storage into 5 ml sterile brain heart infusion medium and incubated at 37°C overnight. The morning after, an OD_600_ of 0.5–0.7 defined a bacterial density of ∼30 × 10^7^ colony‐forming unit (CFU)/ml in the original culture. Slightly anesthetized, age‐, sex‐, and weight‐matched mice were infected with 3.5 × 10^6^ CFU in 25 µl sterile PBS by the intranasal application. Upon infection, mice were continuously monitored for 168 h. To estimate the degree of disease burden and to guarantee a consistent, well‐defined endpoint, weight, temperature, behavior, posture, and appearance of mice were assessed at least every 6 h during the first 3 days. If necessary, additional inspections during the acute phase of infection took place every 3 h. Mice surviving the first 3 days of infection were subsequently controlled at least two times a day according to their health status. Mice losing 15% of their starting weight or displaying a body temperature of less than 34.5°C were killed immediately. One hundred and sixty‐eight hours postinfection, all mice were killed, lungs were harvested after perfusion, and freezed at −80°C for histological examination.

### 2.4 Enzyme‐linked immunosorbent assay

Basal IgM levels were assessed in sera, supernatants of peritoneal washouts, and splenic single‐cell suspensions. To this end, high‐binding 96‐well plates were coated with 5 µg/ml purified anti‐mouse IgM in a coating buffer overnight. The other day, the plate was blocked for 1 h with an enzyme‐linked immunosorbent assay (ELISA) blocking buffer. Samples were diluted in sample buffer and incubated for 2 h on precoated plates at RT. For analysis of PCV13‐, P23‐, or pPS‐specific Ig titers in the sera, plates were coated with either 1 µg PCV13, P23, pPS4, pPS6B, or pPS19F in a coating buffer overnight at 37°C. Before sample incubation, sera were diluted in a sample buffer containing 10 µg/ml cell wall polysaccharides to capture unspecific antibodies. Dilutions were incubated on precoated plates for 3 h at 37°C. To detect TNP–LPS‐induced antibodies, plates were coated overnight with 10 µg/mL NP14‐bovine serum albumin (BSA).

In all cases, the detection was performed using biotinylated anti‐mouse IgM, IgG_1_, IgG_2a_, or IgG_3_ antibody (BD Bioscience) followed by the addition of streptavidin–horseradish peroxidase (HRP) conjugate (Bio‐Techne). HRP‐reaction was induced with 3,3′,5,5′‐tetramethylbenzidine substrate (Thermo Fisher Scientific) and stopped by adding sulfuric acid. Chemoluminescent readout was done at 450/570 nm. All results were normalized to the mean value of Day 0 preimmunization for analysis of the fold changes in specific antibody levels upon immunization.

### 2.5 Cell culture

For in vitro stimulation of splenic cells, CD19^+^ cells were isolated via Magnetic Activated Cell Sorting using anti‐CD19 microbeads and magnetic separation columns (Miltenyi Biotech). Subsequently, enriched B cells were stained with the CellTrace™ Carboxyfluorescein Succinimidyl Ester (CFSE) Proliferation Kit (Invitrogen) at 37°C for 20 min and the reaction was stopped by the addition of complete medium (Roswell Park Memorial Institute medium supplemented with 10% fetal calf serum, 1% l‐glutamine, 1% penicillin/streptomycin (P/S), and 0.05 mM ß‐mercaptoethanol). 2 × 10^6^ CD19^+^ splenic cells were cultured in 24‐well plate inserts in 500 µl medium either unstimulated (US), with 25 µg/ml LPS only or with 25 µg/ml LPS plus 10 ng/ml IL‐4 at 37°C and 5% CO_2_. After 48 h, cells were harvested, washed, and analyzed via flow cytometry. Cell culture supernatants were immediately frozen and stored at −20°C.

### 2.6 LEGENDplex™ (Multi‐Analyte Flow Assay Kit)

To determine the amounts of antibody secreted by isolated splenic B cells in cell culture, the respective supernatants were analyzed with the LEGENDplex™ Mouse Ig Isotyping Panel (BioLegend). To this end, all probes were used both pure and in a 1:5 dilution. The assay was performed in a V‐bottom plate according to the manufacturer's protocol and data acquisition was done using the FACS Canto II flow cytometer (BD Bioscience). BioLegend's LEGENDplex™ Data Analysis Software was applied for analysis (www.biolegend.com/legendplex).

### 2.7 Flow cytometry

For ex vivo analysis of B‐1 cells, 1 × 10^6^ single cells from PL, BM, or spleen were incubated with anti‐mouse CD16/32 (Biolegend) for 15 min on ice for blocking of unspecific Fc‐binding sites. Subsequently, cells were stained with anti‐CD19 FITC, anti‐CD43 PE‐Cy7, anti‐CD5 APC, anti‐IgM APC‐Cy7, and anti‐CD138 PE antibodies (Biolegend and BD Bioscience) for 15 min on ice. To assess cell death, cells were incubated with 7‐aminoactinomycin D (BD Bioscience) before analysis for at least 15 min, but no longer than 60 min.

To analyze proliferation and surface immune globulins after in vitro stimulation, CFSE‐stained cells were incubated with anti‐CD19 V450, anti‐CD43 PE‐Cy7, anti‐CD5 APC, anti‐IgM APC‐Cy7, anti‐IgD‐PerCP, and anti‐IgG_1_ PE (LPS/IL‐4) or with anti‐CD19 APC‐Cy7, anti‐CD43 PE, anti‐CD5 APC, anti‐IgA BV421, anti IgG_2ab_ BB700, and anti‐IgG_3_ PE‐Cy7 (LPS only) (Biolegend and BD Bioscience).

All measurements were performed at the BD FACS Canto II and biaxial gating was done with FlowJO Version 10.

### 2.8 Real‐time PCR

Bone marrow stromal cells (BMSCs) were separated from the pool of total BM cells based on adherence as previously described.^22^ After 48 h of culture in minimum essential medium (Sigma‐Aldrich) supplemented with 10% fetal bovine serum, 1% P/S, 1% l‐glutamine, and 0.05 mM ß‐mercaptoethanol, all cells were harvested by trypsinization and total RNA contents were isolated using the ExtractMe Total RNA Kit (Blirt). Complementary DNA (cDNA) synthesis was done with the TranscriptMe cDNA Kit (Blirt) according to the manufacturer's protocol. Subsequently, real‐time (RT)‐PCR was performed with the Sensi‐fast SYBR No‐Rox Kit (Bioline). For each approach, 20 ng cDNA in 10 µl SYBR mix was applied and ß‐actin was used as a reference gene. RT‐PCR primer was designed via qpcr.probefinder.com and produced by biomers (IL‐9 sequence forward: GCCTCTGTTTTGCTCTTCAGTT/IL‐9 sequence reverse: GCATTTTGACGGTGGATCAT). The RT‐PCR was run in the Light Cycler® 480 (Roche) and an Advanced Relative Quantification was performed with the Light Cycler® 480 software.

### 2.9 Western blot

Splenocytes were either directly subjected to protein lysis in whole‐cell lysis buffer or further processed by CD19‐MACS to isolate a pure fraction of CD19^+^ B cells. Subsequently, CD19^+^ cells were lysed US or after 24 h of in vitro stimulation with 10 ng/ml IL‐4. All protein lysates were diluted in Roti® Load1 (Roth) 4:1 and boiled at 95°C for 5 min before the performance of sodium dodecyl sulfate‐polyacrylamide gel electrophoresis. Ten percent of gels were prepared and loaded with 1 × 10^6^ cells per sample. For band definition, Precision Plus Protein™ Dual Color Standard Marker (Bio‐Rad) was used. After electrophoresis, the proteins were transferred from the gel onto a nitrocellulose membrane by wet blotting. Membranes were blocked in 5% BSA for 1 h at RT and subsequently incubated with rabbit anti‐SAMSN1 antibody (Novus Biologicals) or mouse anti‐GAPDH antibody (HyTest), followed by secondary incubation with HRP‐conjugated anti‐rabbit IgG light chain (Abcam) or Easy Blot anti‐mouse IgG (GeneTex), respectively. Band detection was performed by adding Westar Supernova Luminol‐enhancer solution (7BioScience) and readout in the VersaDoc (Bio‐Rad).

### 2.10 Statistics

For evaluation and graphical illustration of the data, GraphPad Prism Version 7 was utilized. Statistical testing was performed as indicated in corresponding figure legends.

## 3 RESULTS

### 3.1 Increased frequencies of BM‐resident B‐1b cells and enhanced levels of natural IgM in SLy2‐Ko mice

We previously reported decreased frequencies of B‐1 cells and pPS‐specific antibodies in SLy2‐overexpressing mice, identifying SLy2 as an immunoinhibitory adapter protein.^10^ To analyze the impact of SLy2‐deficiency on immune cell subsets and responses, we have generated Ko mice, globally lacking the expression of SLy2 (Figure S1). On the basis of earlier findings, we expected improved B‐1 cell immunity in the absence of SLy2.

**Figure 1:**
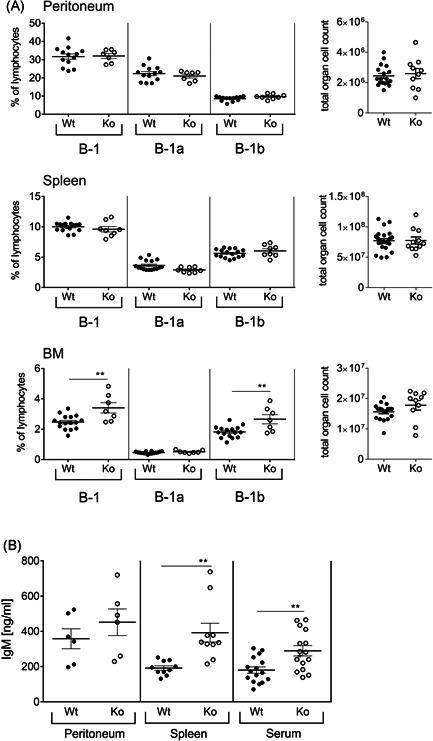
B‐1 cell frequencies and natural IgM titers in SLy2‐Wt and Ko mice at a steady state. (A) Frequency of overall B‐1, B‐1a, and B‐1b cells in peritoneum, spleen, and BM of mice, analyzed via flow cytometry and given as the percentage of all single living lymphocytes. In addition, total cell numbers of all three organs are depicted at the right. B‐1 cells were defined as CD19^+^CD43^+^IgM^+^, with B‐1a cells being CD5^+^ and B‐1b cells being CD5^−^, respectively. Data represent *n* = 7–16 mice pooled from three independent experiments and are shown as mean ± *SEM*. Indicated significances were determined by Student's *t* test and a *p‐*value of less than .05 was considered statistically significant (*p < .05, **p < .01). (B) Natural IgM levels in peritoneum, spleen, and serum of mice at steady state, determined by ELISA and given in ng/ml. Data represent *n* = 6–15 mice per genotype pooled from two to three independent experiments and are shown as mean ± *SEM*. Significance was determined by Student's *t *test and a *p*‐value of less than .05 was considered statistically significant (*p < .05, **p < .01). BM, bone marrow; ELISA, enzyme‐linked immunosorbent assay; IgM, immunoglobulin M; Ko, knockout; Wt, wild‐type

Flow cytometry analysis of T cell populations of the thymus and splenic B‐2 cells revealed equal proportions in SLy2‐Ko mice as compared to Wt littermates. Furthermore, these mice hold normal numbers of dendritic cells, neutrophils, and macrophages/monocytes (Figure S2). In addition, we found the total organ cell counts in peritoneum, spleen, and BM of SLy2‐Ko mice being largely unaltered, accompanied by comparable frequencies of B‐1 cells in peritoneum and spleen (Figure 1A). However, SLy2‐Ko mice displayed an increased proportion of B‐1 cells in the BM, referable to an increase in B‐1b cells with B‐1a cell numbers remaining equal (Figure 1A). Figure S3 exemplarily shows our flow cytometry gating strategy for B‐1 cells, defined as CD19^+^CD43^+^IgM^+ ^and CD5^+/−^.

Since BM‐resident B‐1 cells are assumed to be the main producers of natural protective antibody,^23^ we subsequently examined IgM levels in peritoneal washouts, supernatants of splenic single‐cell suspensions, and serum of mice at steady state. While concentrations of global IgM were comparable in peritoneal washouts of Wt and Ko littermates, they were significantly increased in both, spleen and serum of SLy2‐deficient mice (Figure 1B).

### 3.2 LPS and IL‐4 induced in vitro production of IgG_2b_ is favored in isolated splenic B cells from SLy2‐Ko mice

**Figure 2:**
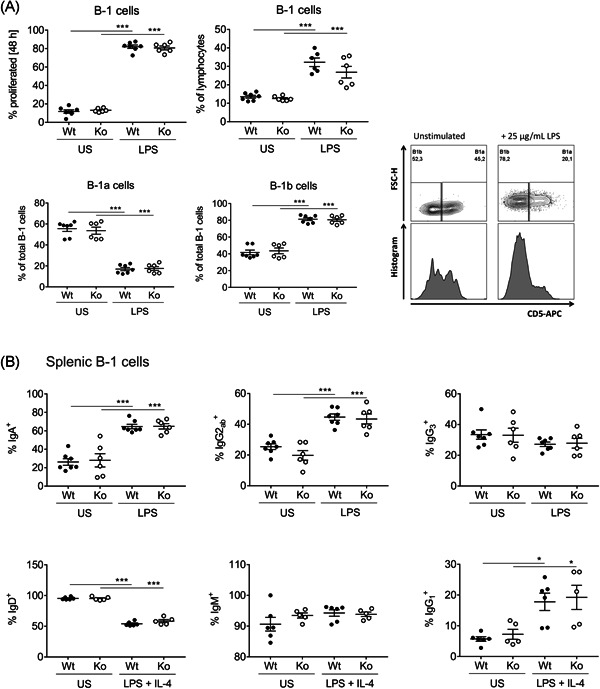
In vitro analysis of proliferation and class switch of isolated splenic B cells upon stimulation with LPS/IL‐4. (A) Isolated splenic B cells were stimulated with 25 µg/ml LPS for 48 h. For assessment of the proliferation rate, CFSE staining was performed before cultivation, and cells were analyzed by flow cytometry after 48 h. Cell ratios are given as a percentage of all single lymphocytes or relative to the whole B‐1 cell population. (B) CFSE‐stained B cells were stimulated with 25 µg/ml LPS only or with LPS + 10 ng/ml IL‐4 for 48 h. Subsequently, class‐switch was investigated by flow cytometry staining of indicated surface immunoglobulins (IgA, IgG_2ab_, IgG_3_, IgD, IgM, and IgG_1_) on all B‐1 cells defined as CD19^+^CD43^+^. Data represent *n* = 6 mice from two independent experiments and error bars depict the mean ± *SEM*. Significance was determined by one‐way analysis of variance with multiple comparisons and a *p‐*value of less than .05 was considered statistically significant (*p < .05, ***p < .001). CFSE, carboxyfluorescein succinimidyl ester; FSC‐H, forward scatter height; IL‐4, interleukin‐4; Ko, knockout; LPS, lipopolysaccharide US, unstimulated; Wt, wild‐type

Proceeding from ex vivo analysis of immune cell populations and natural IgM, we subsequently performed in vitro assays to assess the function and responsiveness of B cells upon stimulation. To this end, splenic B cells were cultured with 25 µg/ml LPS for 48 h or left US as a control. As shown in Figure 2A, the addition of LPS induced extensive proliferation (upper panel left) and a proportional enrichment of CD19^+^CD43^+^IgM^+^ B‐1 cells (upper panel right). Moreover, we observed a significant shift from CD5^+^ B‐1a cells towards CD5^−^ B‐1b cells within the overall B‐1 cell population upon stimulation (Figure 2A, lower panel). This observation is in accordance with a recent publication, proposing the downregulation of surface‐CD5 on B‐1a cells in response to Toll‐like receptor (TLR)‐mediated activation.^24^

Since IgM antibodies have been shown to be increased in both, spleen and serum of SLy2‐Ko mice, we were wondering whether SLy2 might be involved in the regulation of class‐switch events. Therefore, splenic B cells were again stimulated with LPS alone or with LPS + IL‐4 for in vitro induction of class‐switch as previously reported.^25^ Afterwards, the expression of six surfaces Ig on B‐1 cells was examined. As depicted in Figure 2B, LPS‐stimulation significantly induced the expression of surface IgA and IgG_2ab_, whereas IgG_3_ expression remained unaltered (upper panel). Simultaneous addition of LPS and IL‐4 triggered downregulation of surface IgD and upregulation of IgG_1_, while the expression of IgM on the surface remained constant (lower panel).

Complementary, we assessed the concentration of secreted antibodies in these culture supernatants in the absence or presence of stimulation. While levels of secreted IgM, IgG_1_, IgG_2a_, and IgG_3_ were comparable between the genotypes, SLy2‐Ko B cells tended to produce higher amounts of IgG_2b_, with differences being significant upon stimulation with LPS + IL‐4 (Figure S4).

In summary, proliferation, proportional changes within the B‐1 cell population, and surface Ig class‐switch are equally efficient in SLy2‐Wt and Ko B cells derived from the spleen. However, in SLy2‐Ko mice, the production of IgG_2b_ antibodies was favored in all three conditions as compared to Wt controls, with a significant difference in response to addition of IL‐4 (Figure S4).

### 3.3 Increased production of specific IgM antibodies towards TI pneumococcal vaccine in SLy2‐Ko mice

The importance of SLy2 regarding B‐1 cell responses towards pneumococcal antigens has been previously pointed out by the investigation of SLy2‐overexpressing mice in our group. These mice displayed significantly decreased levels of specific antibodies after injection with the pneumococcal vaccine.^10^ Accordingly, we wanted to find out whether SLy2‐deficiency improves the immune response towards pneumococcal immunization.

**Figure 3:**
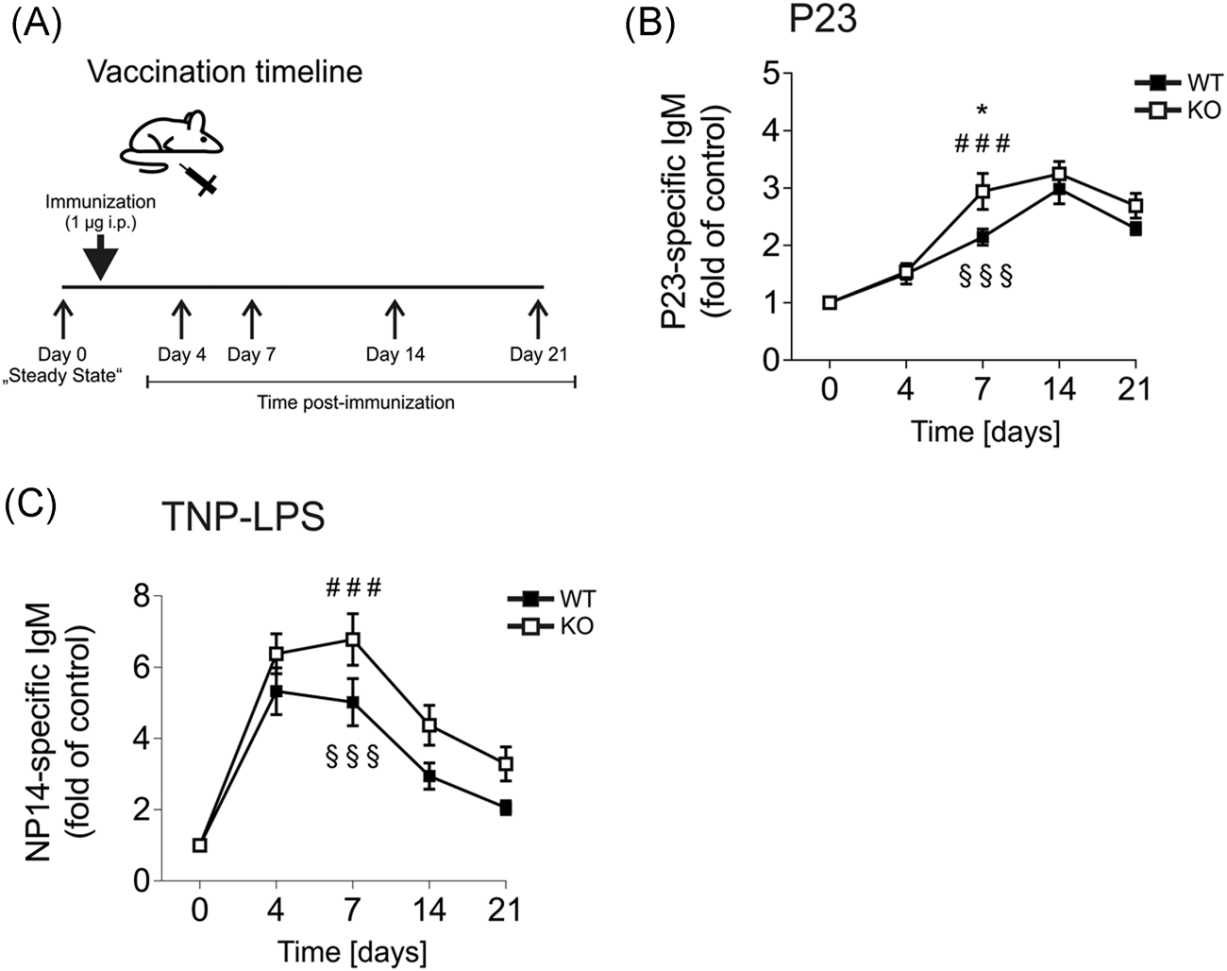
Pneumovax 23 (P23)‐ and TNP–LPS‐specific IgM titers in SLy2‐Wt and Ko mice upon vaccination. (A) Vaccination timeline, depicting the time points of blood sampling: before and 4, 7, 14, and 21 days after immunization. (B) P23‐ and (C) TNP–LPS‐specific IgM responses in the serum of mice after immunization, presented as fold of preimmune titer. Data represent *n* = 7–16 mice per genotype pooled from two independent experiments and are shown as mean ± *SEM*. Significance was determined by a two‐way analysis of variance with multiple comparisons and a *p*‐value of less than .05 was considered statistically significant (*p < .05, **p < .01). IgM, immunoglobulin M; Ko, knockout; LPS, lipopolysaccharide; TNP, trinitrophenyl hapten; Wt, wild‐type

P23 is a pure mixture of 23 pPS serotypes, triggering TI immune responses and leading to the rapid production of IgM.^17^ We immunized SLy2‐Wt and Ko littermates with 1 µg P23 and measured serum IgM levels at 4, 7, 14, and 21 days postimmunization (see vaccination timeline in Figure 3A). Figure 3B illustrates the amount of P23‐specific IgM over time and normalized to day 0 (the fold of control). IgM titers were significantly increased in SLy2‐deficient mice as compared to Wt 7 days postimmunization, indicating an improved TI antibody response upon KO of SLy2 (Figure 3B). Ancillary, mice were immunized with the TI antigen TNP–LPS. Figure 3C shows NP14‐specific serum IgM levels as fold of preimmune titers. Again, SLy2‐deficient mice tended to produce more amounts of specific IgM upon vaccination as compared to their Wt counterparts (Figure 3C).

### 3.4 Enhanced B‐1 cell frequencies and pPS‐specific IgG_2a_‐levels in SLy2‐Ko mice upon immunization with pneumococcal conjugate vaccine

**Figure 4:**
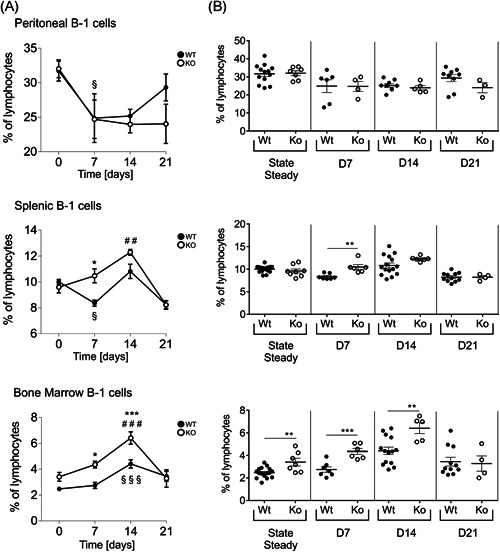
Frequency of B‐1 cells in the peritoneum, spleen, and BM of mice before and after injection with Prevenar 13. (A) Over time progression of B‐1 cell frequencies in SLy2‐Wt and Ko mice from Day 0 preimmunization to Days 7, 14, and 21 postimmunization. Animals were killed at all four‐time points for collection of peritoneal washouts, spleen, and BM. Cell percentages are given related to all single living lymphocytes and represent *n* = 4–16 mice per genotype pooled from two to four independent experiments. Error bars indicate the mean ± *SEM* and significance was determined by two‐way analysis of variance with multiple comparisons. “§” indicates the significance of Wt curves and “#” of Ko curves in comparison to Day 0, and asterisks reflect significant differences between the genotypes. A *p‐*value of less than .05 was considered statistically significant (§, #, *p < .05; §§, ##, **p < .01; §§§, ###, ***p < .001). (B) Direct comparison of B‐1 cell frequencies at respective days, shown as dot plots and given as a percentage of all single living lymphocytes. Data represent *n* = 4–16 mice per genotype pooled from two to four independent experiments. Error bars indicate the mean ± *SEM* and significance between the genotypes was determined by Student's *t* test. A *p*‐value of less than .05 was considered statistically significant (**p < .01, ***p < .001). BM, bone marrow; Ko, knockout; LPS, lipopolysaccharide; Wt, wild‐type

Immunization with P23 successfully elicits immune responses in healthy adults. Nevertheless, it has been shown to provide only limited and temporally transient protection in high‐risk groups which is problematic, since especially infants, the elderly and immunocompromised patients suffer from increased incidence of IPD.^26^ Thus, the conjugate‐vaccine PCV13 was introduced and is routinely recommended for high‐risk candidates. It contains 13 pPS‐serotypes coupled to a carrier protein, thereby additively inducing thymus/T‐cell‐dependent (TD) responses, providing reinforced protection.^27^ As we were interested in the response of SLy2‐Ko mice towards the TD vaccine, we immunized them with 3 µg PCV13 and subsequently investigated serum Ig responses and B‐1 cells in three different organs at 7, 14, and 21 days postimmunization. The percentages of B‐1 cells are presented as curves over time (Figure 4A) and as dot plots for direct comparison of Wt and SLy2‐deficient individuals (Figure 4B).

As a consequence of PCV13 injection, peritoneal B‐1 cell frequencies markedly declined in both genotypes after 7 days, most likely attributable to the emigration of activated B‐1 cells from the peritoneum (Figure 4, upper panel). While rates of splenic B‐1 cells were irregular in Wt animals, they increased in Ko mice over time, reaching significance after 14 days as compared to preimmune conditions (Figure 4, middle panel). Percentages of BM‐resident B‐1 cells significantly increased over time in both, Wt and Ko littermates, with B‐1 cell proportions being substantially higher in Ko mice 7 and 14 days postimmunization (Figure 4, lower panel).

Considering the profound alterations in B‐1 cell frequencies, we next measured concentrations of IgM and IgG antibodies, specifically produced upon exposure to PCV13. PCV13‐specific antibody levels are depicted as fold of preimmune titers over time in curves (Figure 5A) or as dot plots for direct comparison of genotypes (Figure 5B). Immunization rapidly induced significant levels of specific IgM from Day 7 postvaccination on, with titers being identical in both genotypes (Figure 5A,B). Conjugate immunization also stimulated the production of IgG_1_ and IgG_3_ starting after 14 days, indicating the formation of a germinal center response and class‐switch events (Figure 5A,B).^28^ SLy2‐Ko mice tended to produce fewer amounts of IgG_1_ and more IgG_3_ than Wt littermates, but those differences did not reach statistical significance.

Figure 5Prevenar 13 (PCV13)‐specific IgM and IgG in the serum of mice upon immunization. (A) Over time progression of PCV13‐specific IgM, IgG_1_, IgG_2a_, and IgG_3_ levels in the serum of mice from Day 0 preimmunization to Days 7, 14, and 21 postimmunization. All values are given as fold of preimmune titers and were assessed within two to four independent experiments per day. Data represent *n* = 4–16 mice and error bars depict the mean ± *SEM*. Significance was determined by a two‐way analysis of variance (ANOVA) with multiple comparisons. “§” indicates the significance of Wt curves and “#” of Ko curves in comparison to Day 0, and asterisks reflect significant differences between the genotypes. A *p*‐value of less than .05 was considered as statistically significant (§, #, *p < .05; §§, ##, **p < .01; §§§, ###, ***p < .001). (B) Direct comparison of PCV13‐specific Ig‐titers in SLy2‐Wt and Ko mice. Data are shown as dot plots and significances within genotypes at different time points were assessed by Student's *t* test. A *p*‐value of less than .05 was considered statistically significant (*p < .05). (C) Over time progression of pPS3, pPS4, pPS6B, and pPS19F‐specific IgG_2a_ serum antibody‐titers (fold of Day 0). Data represent *n* = 9–16 mice and error bars indicate the mean ± *SEM*. Significance was determined by one‐way ANOVA with multiple comparisons. “#” indicates the significance of Ko curves in comparison to day 0, and asterisks reflect significant differences between the genotypes. A *p*‐value of less than .05 was considered statistically significant (#, *p < .05; ##, **p < .01; ###, ***p < .001). IgM, immunoglobulin M; Ko, knockout; pPS, pneumococcal polysaccharide; Wt, wild‐type

**Figure:**
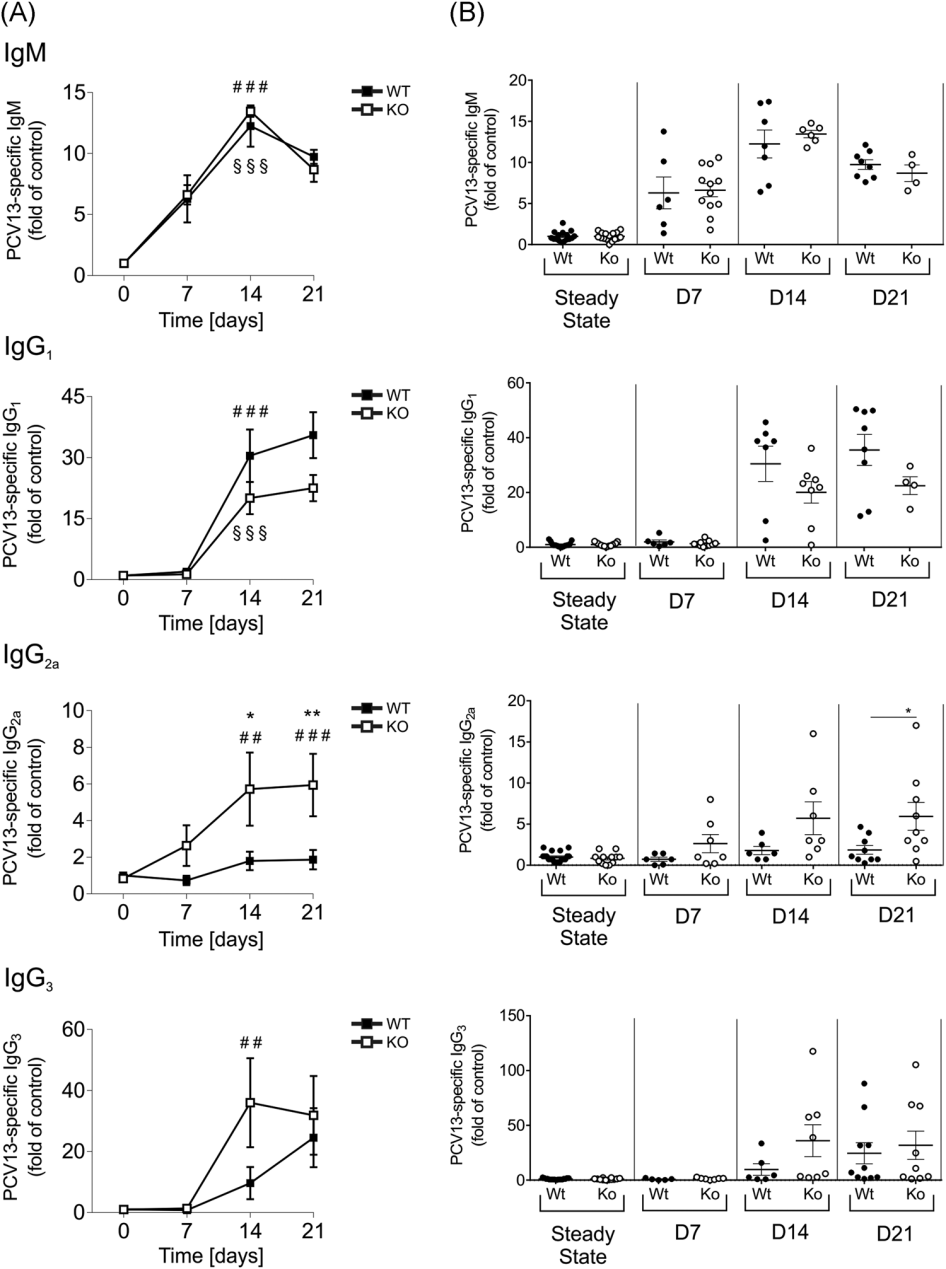

**Figure:**
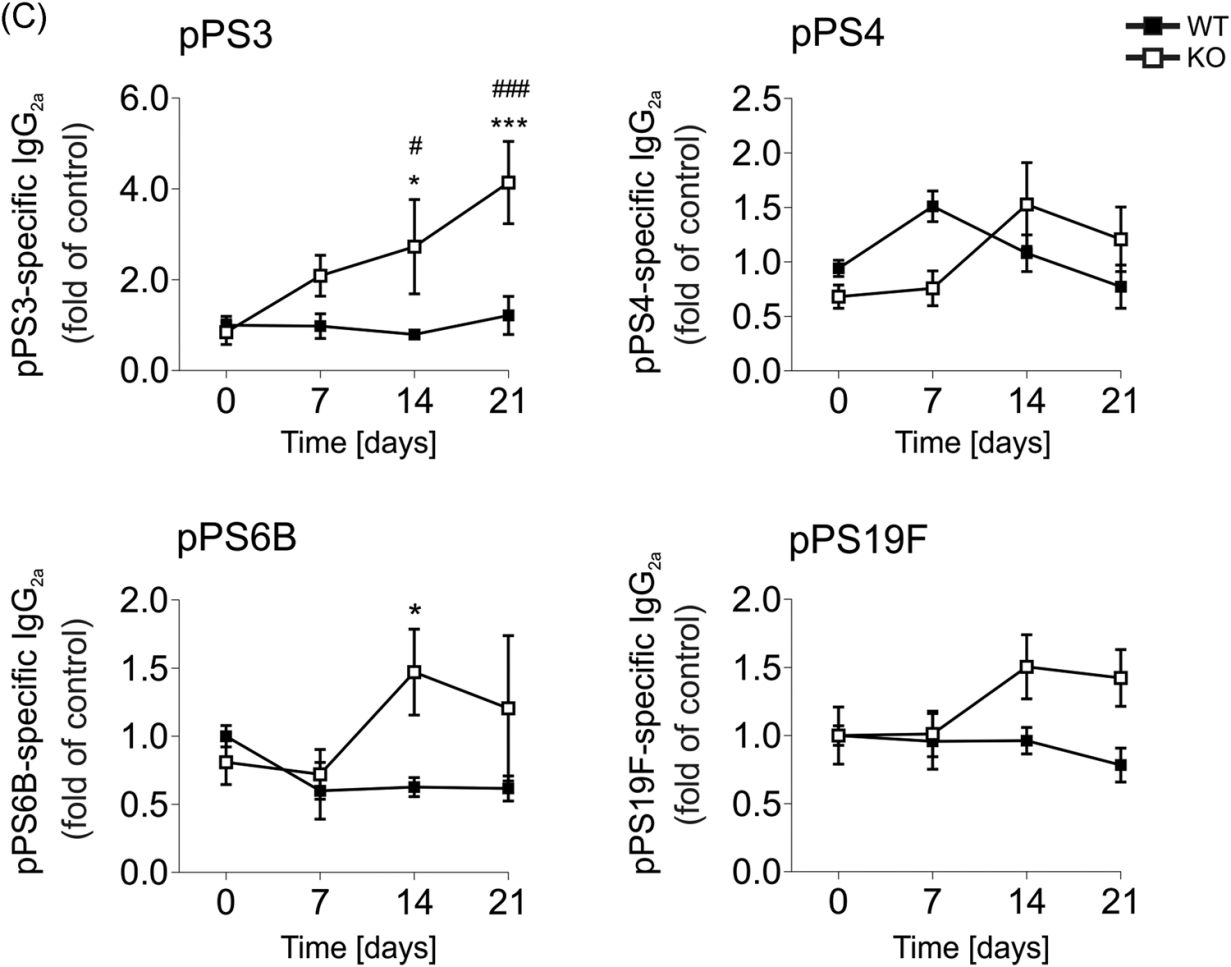

While the amounts of IgG_2a_ produced by Wt animals were negligible, it seemed to be strongly favored in some SLy2‐deficient individuals. As illustrated in Figure 5A,B, PCV13‐specific IgG_2a_ was produced in Ko mice from Day 7 on, reaching significance at Days 14 and 21 postvaccination. Differences between the genotypes were statistically significant. Complementarily, we evaluated IgG_2a_ titers against the pneumococcal serotypes 3, 4, 6B, and 19F, all of which are highly virulent serotypes of *S. pneumoniae*, frequently found in clinical contexts. We found increased serum concentrations of pPS3‐, 6B‐, and 19F‐directed IgG_2a_ antibodies in Ko mice after 14 and 21 days. These differences were significant for pPS3 and 6B (Figure 5C).

### 3.5 Unaltered survival rate of SLy2‐Ko mice in the course of acute pneumococcal lung infection

**Figure 6:**
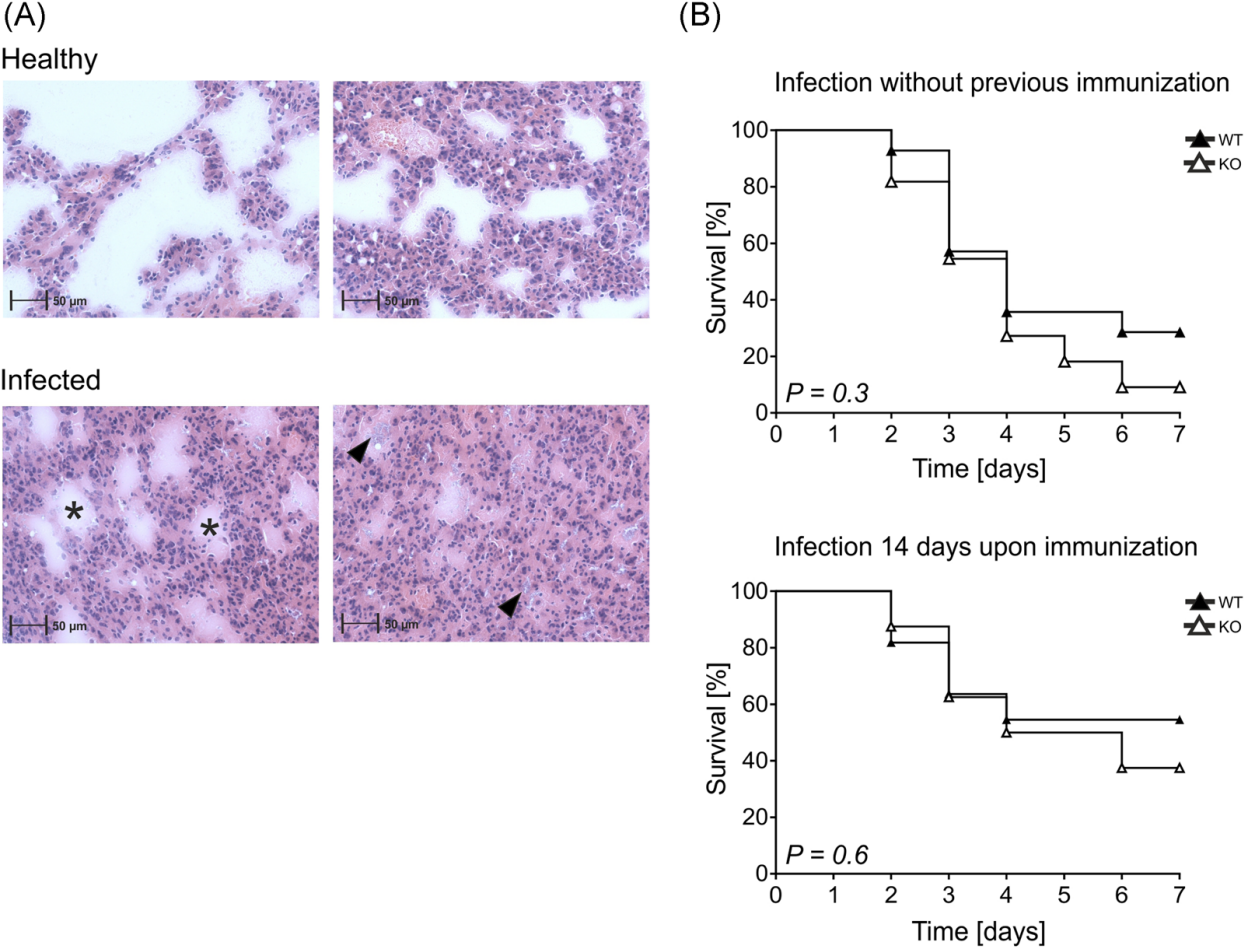
Histology and survival of SLy2‐Wt and Ko mice upon intranasal challenge with *Streptococcus pneumoniae* serotype 3. (A) Exemplary histological images of lung tissue from one healthy mouse (pictures on the top) and one sick mouse (pictures on the bottom) (magnitude ×100). Lungs were shock‐frosted in liquid nitrogen upon perfusion with PBS and stored at −80°C. For all four pictures, 12 µm cryosections of lung tissues were performed and stained with hematoxylin and eosin solution. The asterisks mark protein‐rich edema within alveolar spaces and arrowheads indicate immune cell infiltrations as visualized by their blue‐stained nuclei. (B) Seven days survival analysis of SLy2‐Wt and Ko mice upon intranasal infection with 3.5 × 10^6^ CFU/mouse without preceding immunization or after intraperitoneal vaccination with 1 µg P23 14 days before infection. Data are shown in Kaplan–Meier survival graphs and represent *n* = 14 mice per genotype pooled from three independent infection experiments. Indicated significances were determined using the logrank (Mantel–Cox) test. CFU, colony‐forming unit; Ko, knockout; PBS, phosphate‐buffered saline; Wt, wild‐type

Proceeding from the marked differences in antibody responses towards P23 and PCV13, we decided to assess the survival rate of Wt and Ko animals during acute pneumococcal infection. Since the ELISAs revealed the most pronounced differences for pPS3, we decided to use *S. pneumoniae* serotype 3 for our infection experiment. Mice were intranasally challenged with 3.5 × 10^6^ CFU and subsequently monitored for 7 days. The burden of disease was estimated using precise physiological criteria (e.g., weight, temperature, and appearance) to guarantee constant and ethical endpoint definition. 24–72 h upon challenge, mice developed symptoms of the disease including dropping temperature, fast breathing, and weight loss. Intranasal application of *S. pneumoniae* induced pronounced symptoms of pneumonia such as severe consolidation of the lung tissue as compared to healthy controls. Figure 6A exemplarily shows hematoxylin and eosin (HE)‐stained lung cryosections of one healthy mouse (top), and one sick mouse (bottom), with the latter being killed during acute infectious disease. *S. pneumoniae*‐infection induced intra‐alveolar accumulation of protein‐rich fluids, visualized as light‐pink material in the HE‐staining (indicated by asterisks, Figure 6A). Moreover, we could observe immune cell infiltration into the infected tissue, as visible by blue‐colored nuclei within alveolar spaces (indicated by arrowheads, Figure 6A).

As shown in Figure 6B, WT and KO mice were infected in parallel either without preceding immunization (upper panel) or after P23‐vaccination (lower panel). We could not detect significant differences between the survival rates of Wt and SLy2‐deficient mice during acute infection. The injection of P23 14 days before challenge improved overall survival in both experimental groups, indicating that immunization conferred immune protection in a genotype‐independent manner (Figure 6B, lower panel).

To sum up, we found increased vaccine‐specific antibody responses in SLy2‐deficient mice in the context of both, TI and TD pneumococcal vaccination. However, these were not sufficient to improve the survival of mice during acute pneumococcal pneumonia.

## 4 DISCUSSION

*S. pneumoniae* is a major bacterial pathogen in humans, being responsible for a huge burden of disease worldwide. One of the most frequent manifestations of pneumococcal infection is pneumonia, accounting for around 1 million deaths of children annually.^29^, ^30^, ^31^ Beyond its substantial contribution to child mortality, *S. pneumoniae* also represents a significant health problem in the elderly and immune‐deficient patients.^17^, ^30^, ^32^ By secretion of toxic mediators, *S. pneumoniae* efficiently induces host inflammation and evades the immune system through a variety of mechanisms.^16^ The outer pPS capsule is the most immunogenic structure of *Pneumococci* and nowadays, more than 90 different serotypes are known.^26^ P23 and PCV13 cover 23 and 13 of the most virulent and prevalent serotypes of *S. pneumoniae*, respectively. However, many patients do not mount or sustain adequate levels of pPS‐specific antibodies upon immunization. Thus, severe clinical manifestations of pneumococcal infection remain common despite vaccination.^21^, ^33^

Existing studies independently highlight the importance of innate B‐1 cells in the context of infection caused by microbial pathogens such as *S. pneumoniae*.^34^, ^35^ Particularly B‐1b cells represent an important first‐line defense upon pneumococcal infection by the production of pPS‐specific antibodies. They are substantially involved in the response towards a pneumococcal vaccine and were shown to mediate long‐lasting immunity against *S. pneumoniae*.^14^, ^15^

Here, we demonstrate increased frequencies of B‐1b cells in the BM of SLy2‐deficient mice (Figure 1A). The enrichment in BM B‐1b cells is likely to explain the significantly elevated levels of circulating IgM measured in both, serum and spleen of these mice (Figure 1B). Conveniently, B‐1 cells of the BM have been identified as the major source of spontaneously secreted IgM in the absence of antigen.^23^ The BM is a well‐known niche for antibody‐secreting cells, providing survival factors and allowing the deposition of immune globulin into the blood.^36^, ^37^ Recently, IL‐9 has been reported to be an important growth factor specifically for B‐1b cells and it is known to be produced in the BM.^38^, ^39^ Since increased production of IL‐9 could drive survival and expansion of B‐1b cells in SLy2‐deficient mice, we analyzed IL‐9 expression levels in isolated BMSCs. We observed a tendency of SLy2‐Ko mice to produce more IL‐9, however not in a statistically significant manner (Figure S5).

Notably, while we observed unaltered numbers of peritoneal B‐1 cells, a previous report by Wang and colleagues revealed increased rates of B220^low^/CD5^+^ or IgM^+^/CD5^+^ peritoneal B‐1a cells SLy2‐Ko mice.^40^ Importantly, here we defined B‐1 cells by the simultaneous surface expression of CD19, CD43, and IgM, as previously published by Baumgarth.^11^ The profound differences in B‐1 cell phenotype definition explain the divergence of results between these two studies. When we performed additional staining using solely IgM, B220, and CD5, we also found increased percentages of B220^+^/IgM^+^ CD5^+^peritoneal B‐1a cells in our mouse model (data not shown).

Following ex vivo analysis of cell populations, we wanted to address whether the proliferation of SLy2‐deficient B cells may be altered in response to TLR engagement. In vitro LPS‐stimulation revealed a similar proliferative capacity of both Wt and Ko cells, implicating that SLy2 is not involved in B‐1 cell LPS‐sensing pathways such as TLR‐4‐dependent signaling.^41^ Since B‐1 cells are known to respond towards TLR‐9‐ligands, we further investigated the proliferation of peritoneal and splenic B‐1 cells from Wt and Ko mice upon CpG stimulation (Figure S6).^42^, ^43^ However, there were no differences seen between the genotypes, supporting the assumption that SLy2 might be dispensable for functional TLR‐signaling in B‐1 cells.

On the other hand, the enhanced secretion of IgG_2_ antibodies by isolated splenic B cells from SLy2‐Ko mice indicates an intrinsic function of the adapter for IgG_2_‐isotype formation. The given differences only reached statistical significance upon IL‐4‐addition (Figure S4). Since IL‐4 is the main cytokine produced by T follicular helper cells in the course of the germinal center reaction, these results indicate a specific role of SLy2 not only for TI responses but also for the generation of IgG_2b_ antibodies in cooperation with T‐cell help.^44^

Upon immunization with the pPS vaccine P23, Ko mice produced elevated levels of specific IgM (Figure 3B). This observation matches to the dampened immune responses towards P23 recently found in SLy2‐Tg mice.^10^ Thus, these data collectively strengthen the hypothesis of SLy2 as an inhibitor of B‐1 cell‐mediated antibody production against pPS in a TI setting.

On the basis of these findings, we got also interested in the responsiveness of SLy2‐Ko mice towards pneumococcal vaccine under TD conditions. As a consequence of the ip injection of PCV13, B‐1 cells in the peritoneum markedly declined. This drop was attributable to a rapid and significant reduction of B‐1a cells over time, with the B‐1b cell population being increased after 21 days (data not shown). At the same time, we observed an enrichment of B‐1 cells in the BM with cell percentages being significantly higher in Ko mice (Figure 4A,B). It is known that activated peritoneal B‐1 cells rapidly migrate to the omentum and peripheral lymphoid tissues to produce and spread protective antibodies.^45^, ^46^ Therefore, our observation points to the efficient activation of peritoneal B‐1 cells through the administration of PCV13. Whether the continuous increase in BM‐resident B‐1 cells upon vaccination is attributable to BM homing or merely a result of local proliferation, is a question of high interest that remains to be solved.

Investigation of PCV13‐specific serum Ig revealed a fast induction of IgM in both SLy2‐Wt and Ko animals on a highly comparable level. This is in contrast to what we have found earlier regarding P23, indicating that SLy2 might not be involved in the regulation of IgM responses under TD conditions.

Moreover, we demonstrate that SLy2‐deficiency specifically favored the production of PCV13‐ and pPS‐specific IgG_2_ antibodies, which is in accordance with the increased production of IgG_2_ upon IL‐4 stimulation previously observed in vitro (Figures 4 and S4). Of all four IgG subclasses known, IgG_2_ is the one that is primarily induced in response to polysaccharide antigens.^47^, ^48^ Interestingly, phenotypic manifestations of human Trisomy 21 include impaired responses towards a pneumococcal vaccine and a specific lack of IgG_2_.^49^, ^50^ Since SLy2 is overexpressed in peripheral blood cells of patients with DS, our data allow the suggestion that excessive SLy2 might interfere with the formation of proper IgG_2_ responses in DS patients.

*S. pneumoniae* infection experiments did not reveal any difference in survival of SLy2‐Ko mice as compared to Wt, suggesting that increased levels of natural and pPS‐specific antibodies were not sufficient to induce survival advantages. It is well‐established that the outcome of infectious pneumonia depends on the proper activation of neutrophils, alveolar macrophages (AM) and natural killer  cells.^51^, ^52^ For example, disruption of AM function has been reported to increase susceptibility and mortality of mice in the context of *S. pneumoniae* infection.^53^, ^54^ However, differential expression of SLy2 only affects B‐1 cells, but not T cell or myeloid cell populations (Figure S2).^10^, ^40^ When assessing the bacterial blood burden in our mice 24 or 48 h postinfection, we detected only low to zero numbers of CFUs (data not shown), suggesting that the survival outcome in our infection model mainly relied on the effectiveness of cell‐mediated immune responses in the lung. Possible benefits through increased antibody titers might be undermined by a predominance of antibody‐independent immune mechanisms in our infection model. Yet, the impact of SLy2‐deficiency during systemic pneumococcal infection (e.g., during sepsis) remains to be investigated.

## 5 CONCLUSION

To sum up, our study demonstrates improved pPS‐specific antibody responses in the absence of SLy2 expression in mice. While knocking out SLy2 reinforced IgM responses under TI conditions, it specifically promoted IgG_2_ production in the context of TD conjugate vaccination; suggesting that the role of the adapter protein highly depends on the type of B‐cell stimulation that is given. The in vitro analysis of antibody secretion further points to a B‐cell intrinsic role of SLy2 in the formation of IgG_2 _responses upon TD stimulation via IL‐4.

Considering the suboptimal immune response of many patients towards both, TI and TD pneumococcal immunization, the elevated production of pPS‐specific IgM and IgG_2_ as a result of SLy2‐deficiency is of utmost interest. Differential expression levels of SLy2 in humans could at least in part explain the huge differences seen in the formation of antibody responses towards pneumococcal antigens. Thus, SLy2 should be considered as a potential target for future therapeutic interventions, aiming to improve pneumococcal vaccine‐induced B cell immunity.

## CONFLICT OF INTERESTS

The authors declare that there are no conflict of interests.

## AUTHOR CONTRIBUTIONS

Jennifer Jaufmann designed and carried out the experiments, analyzed the data, and wrote the manuscript. Leyla Tümen, Fee Schmitt, Daniel Schäll, and Max von Holleben performed experiments. Sandra Beer‐Hammer designed experiments, provided experimental and conceptual advice, and wrote the manuscript. All authors discussed the data and edited the manuscript.

## Supporting information

Supplementary information.

Supplementary information.

Supplementary information.

Supplementary information.

Supplementary information.

Supplementary information.

Supplementary information.

## Data Availability

The data that support the findings of this study are available from the corresponding author upon reasonable request.
