# Supplementary figures and images for: SLy2‐deficiency promotes B‐1 cell immunity and triggers enhanced production of IgM and IgG2 antibodies against pneumococcal vaccine
Source: Immun Inflamm Dis. 2020 Oct 24;8(4):736–52. doi: 10.1002/iid3.365 (PMC7654406; doi:10.1002/iid3.365)

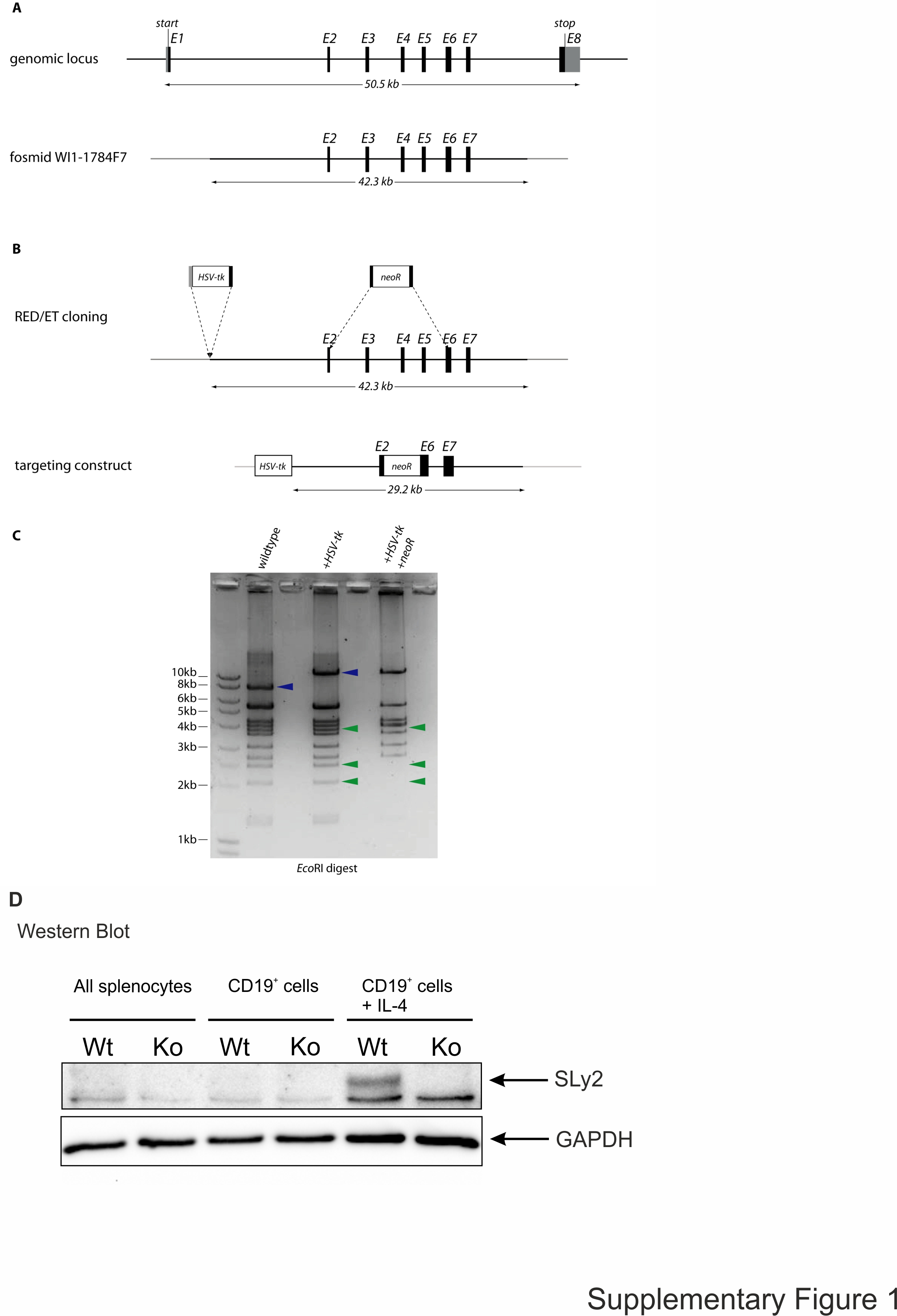

Supplement: Supplementary file 1 — Supplementary information. [file IID3-8-736-s001.jpg]

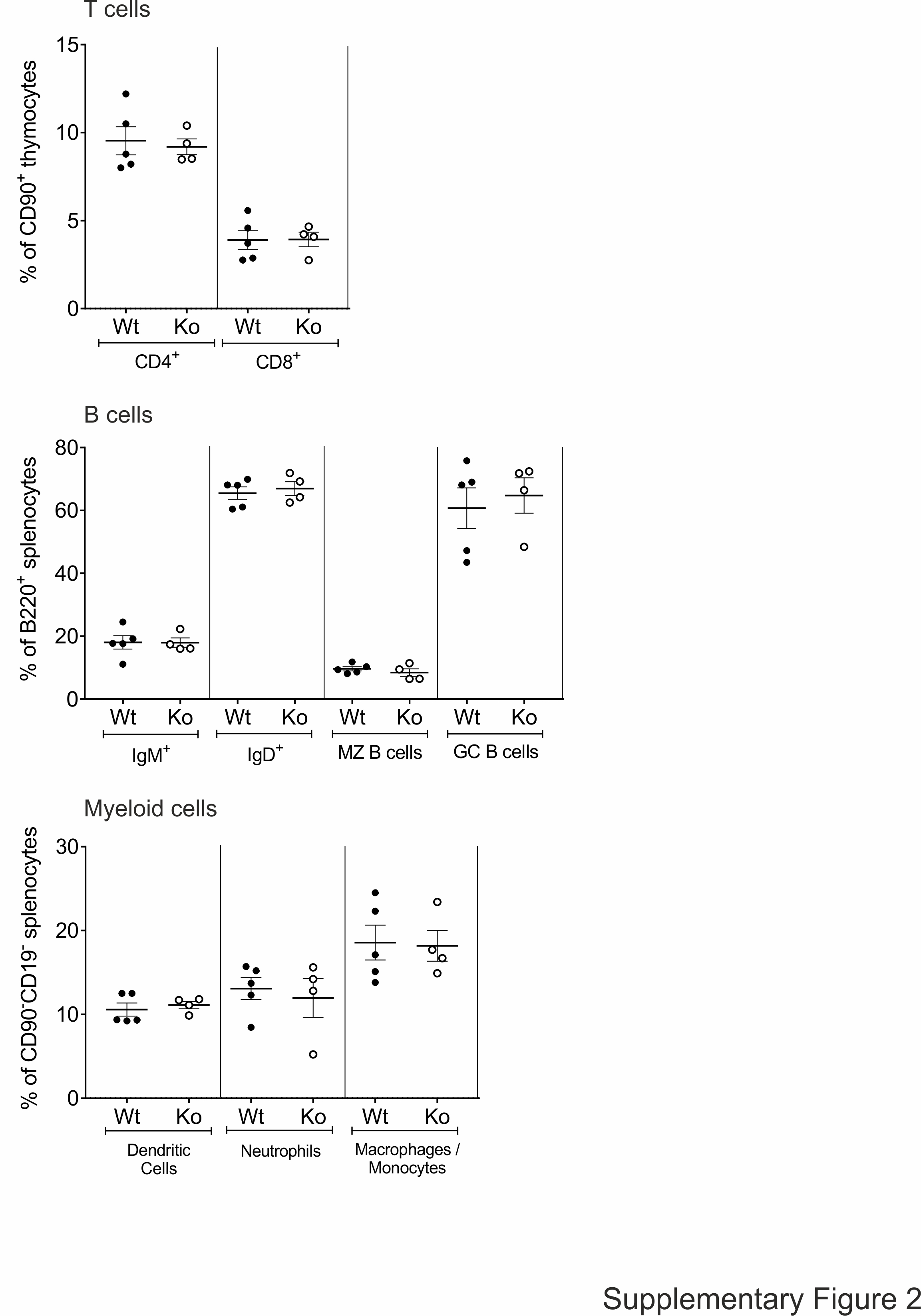

Supplement: Supplementary file 2 — Supplementary information. [file IID3-8-736-s002.jpg]

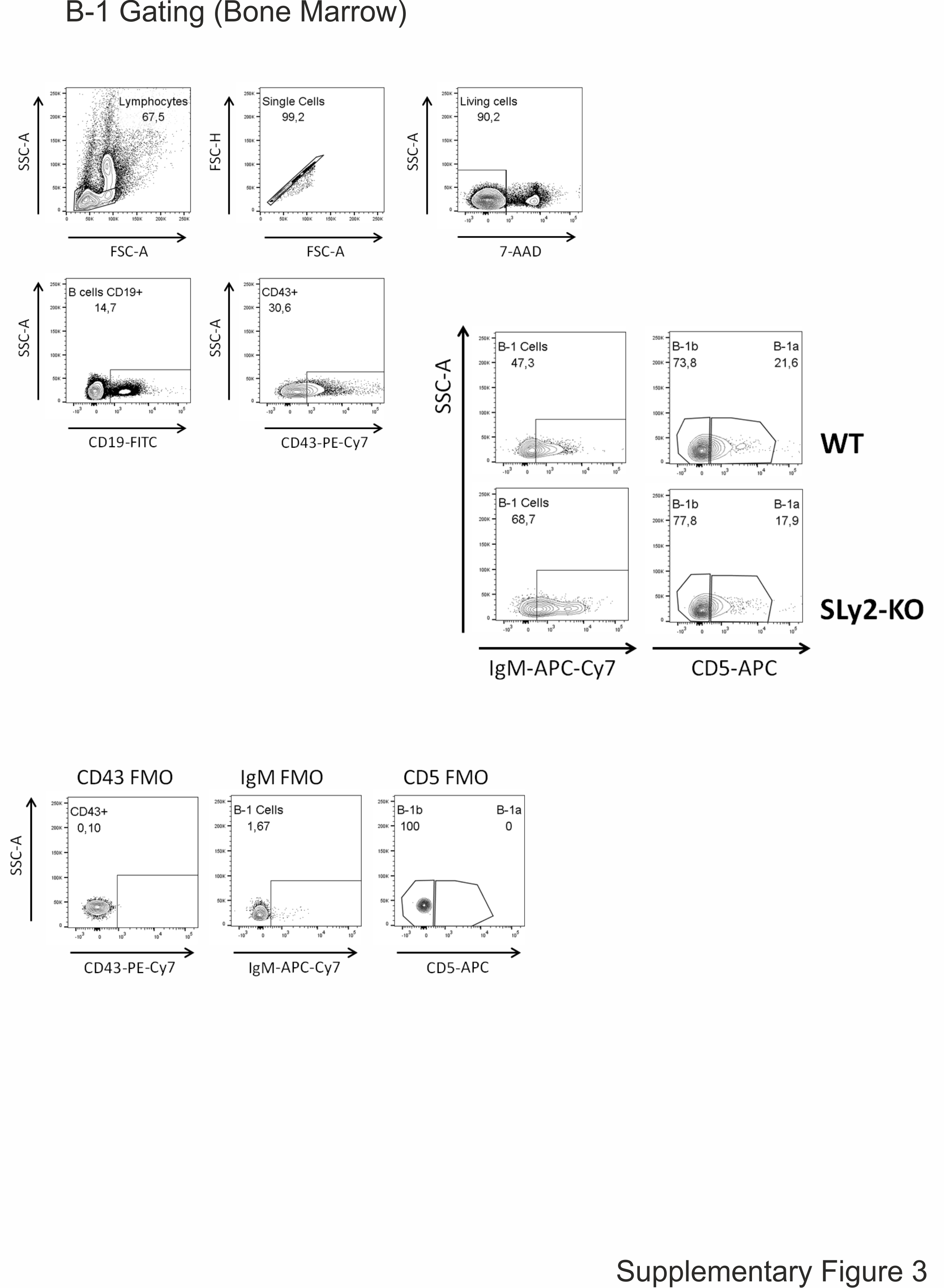

Supplement: Supplementary file 3 — Supplementary information. [file IID3-8-736-s003.jpg]

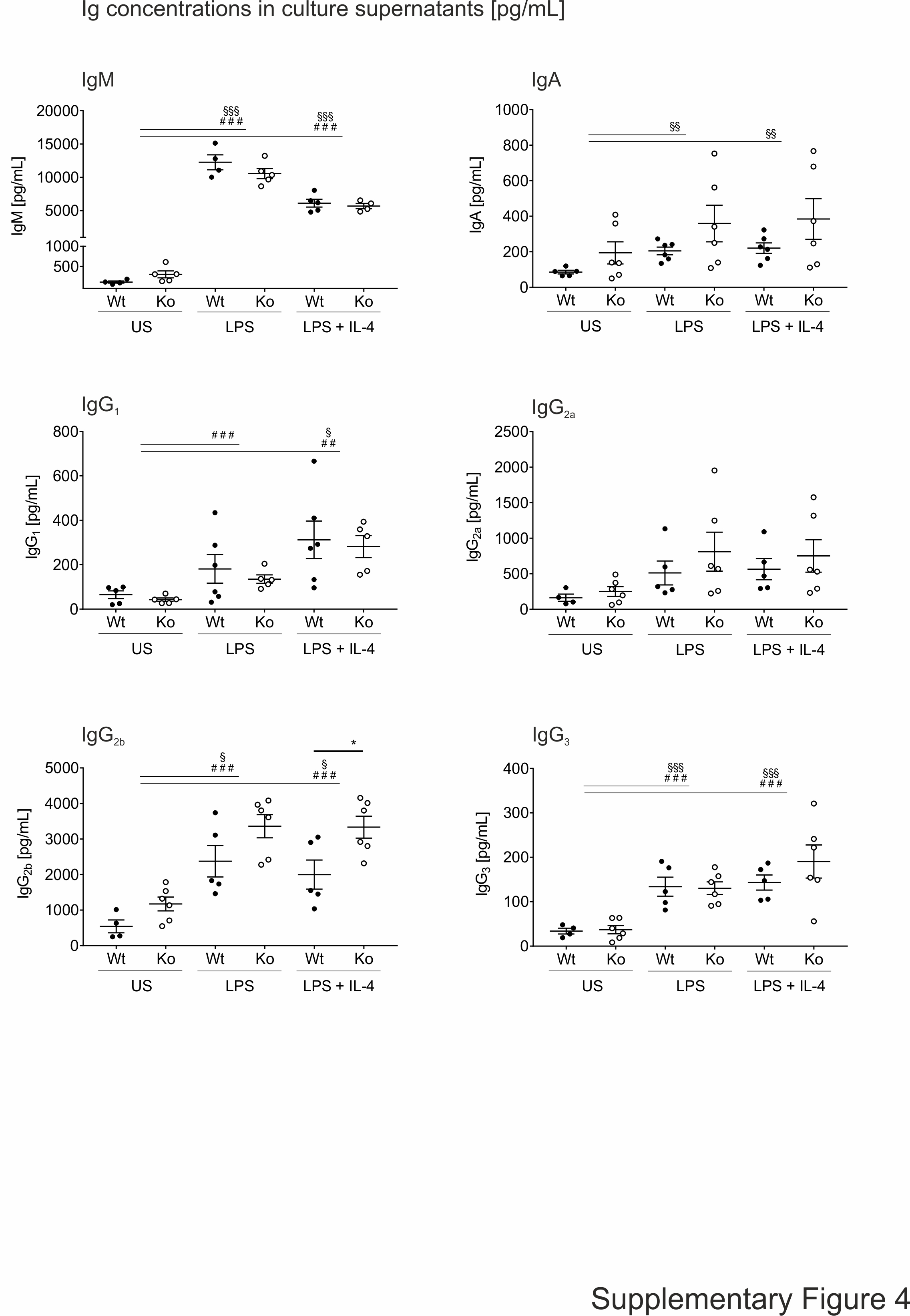

Supplement: Supplementary file 4 — Supplementary information. [file IID3-8-736-s004.jpg]

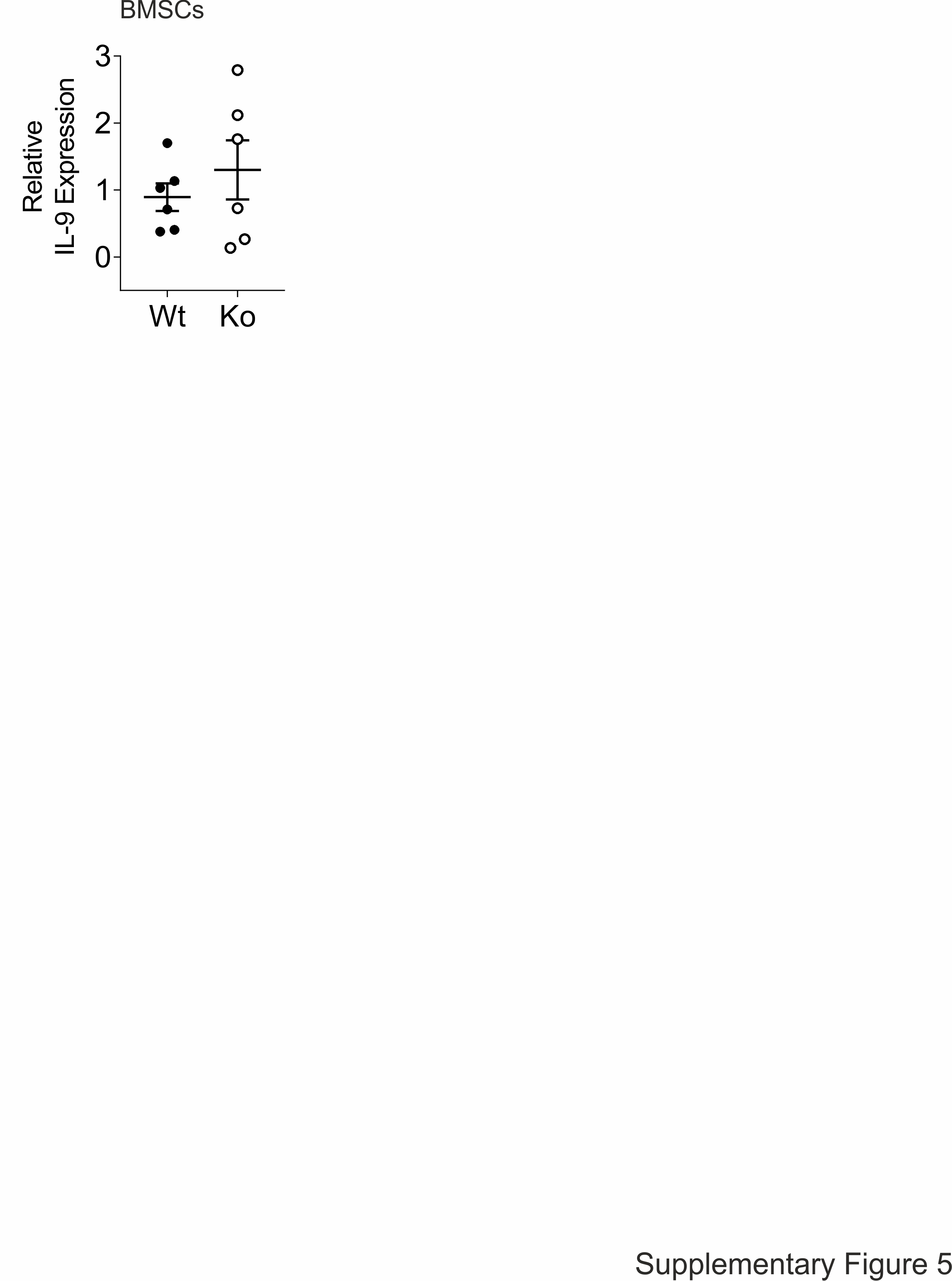

Supplement: Supplementary file 5 — Supplementary information. [file IID3-8-736-s005.jpg]

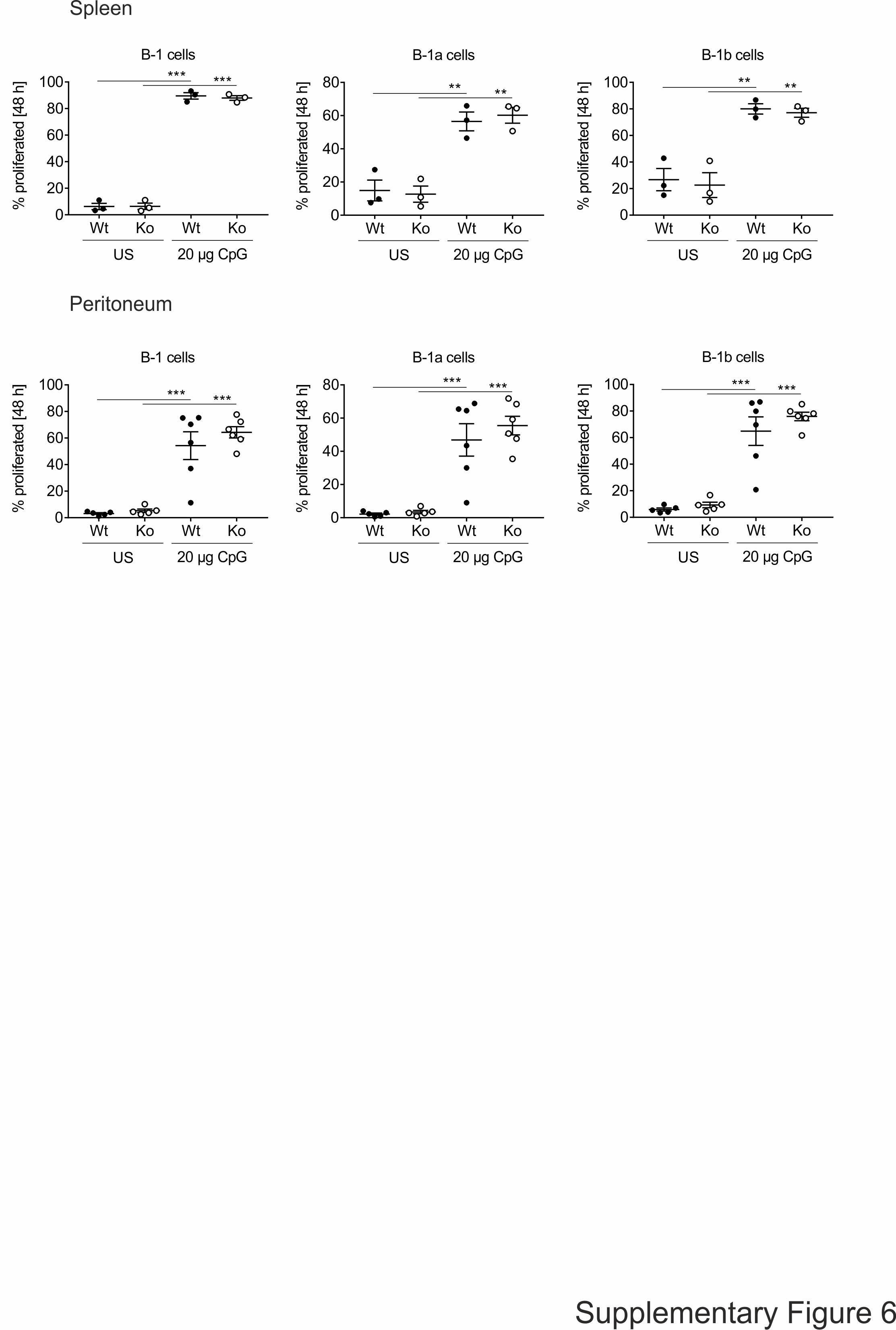

Supplement: Supplementary file 6 — Supplementary information. [file IID3-8-736-s006.jpg]
