# Supplementary material for: SLy2‐deficiency promotes B‐1 cell immunity and triggers enhanced production of IgM and IgG2 antibodies against pneumococcal vaccine
Source: Immun Inflamm Dis. 2020 Oct 24;8(4):736–52. doi: 10.1002/iid3.365 (PMC7654406; doi:10.1002/iid3.365)
Supplement: Supplementary file 7 — Supplementary information. [file IID3-8-736-s007.docx]

Supplementary Figure 1: The Targeting Construct for Generation of SLy2-deficient Mice.

**(A)** The genomic locus of SLy2 and the corresponding fosmid-clone. **(B)** The targeting construct was generated by Red/ET cloning in two consecutive steps. First the HSV-tk cassette was inserted at the border between the genomic sequence and the fosmid backbone. Subsequently, the neoR cassette was integrated, deleting approximately 15kb of genomic sequence from exon2 to 6. Shown above are the fosmid map and both cassettes with their respective arms of homology as generated by PCR prior to recombination. **(C)** Restriction digests showing the correct integration of both selection markers. Blue arrowheads indicate the fragments, which increases in size between the wildtype and the first recombination step due to the incorporation of the HSV-tk cassette. Green arrowheads mark the bands which disappear due to the replacement of the genomic sequence by the neoR cassette. The fosmid WI1-1784F7 was purchased through *www.bacpac.chori.com.* **(D)** Confirmation of the knockout via western blot. Protein lysates from pure splenocytes, unstimulated splenic CD19^+^ cells and IL-4-stimulated CD19^+^ cells are shown. SLy2 is upregulated in B cells upon stimulation and is absent in SLy2-Ko mice. The SLy2 band was detected at ~50 kDa and the GAPDH-loading control at 37 kDa.

Supplementary Figure 2: Thymic T Cells, splenic B-2 Cells and myeloid Cell Populations of SLy2-deficient and Wt littermate Mice.

Cell populations were analyzed at steady state via flow cytometry. T cells of the thymus were defined as CD90^+^TCRᵧδ^-^ and CD4^+^ or CD8^+^ (upper panel). B220^+^ B-2 cells of the spleen were subdivided into IgM^+^, IgD^+^, CD21^+^CD23^-^ marginal zone (MZ) B cells and CD23^+^PNA^+^ germinal centre (GC) B cells (middle panel). Dendritic cells were defined as CD90^-^CD19^-^CD11c^+^, neutrophils as CD90^-^CD19^-^Ly6G^+^CD11b^+^ and macrophages/monocytes as CD90^-^CD19^-^Ly6G^-^CD11b^+^ (lower panel). Dot plots show n=4-5 mice per genotype and data are presented as mean ± SEM.

Supplementary Figure 3: Gating Strategy for Identification of B-1 Cells via Flow Cytometry.

First, single lymphocytes were gated by using the SSC-A, FSC-A and FSC-H parameters. Subsequently, dead cells were excluded by positive selection of 7-AAD^-^ cells. B-1 cells were defined as CD19^+^CD43^+^IgM^+^ and further subdivided into B-1a and B-1b cells, being CD5^+^ and CD5^-^, respectively. Gates were placed according to respective fluorescence minus one (FMO) controls. This B-1 cell gating strategy was equally performed for every organ using organ-specific FMO controls. During the assessment and analysis of data via flow cytometry we have adhered to the “Guidelines for the use of ﬂow cytometry and cell sorting in immunological studies” ([55](#_ENREF_55)).

Supplementary Figure 4: Evaluation of secreted Immunoglobulines in Culture Supernatants of isolated splenic B Cells.

Isolated splenic B cells were stimulated with 25 µg/mL LPS only or with LPS + 10 ng/mL IL-4 for 48 hours. For every mouse, an unstimulated control was performed (US). Subsequently, the levels of IgM, IgA, IgG_1_, IgG_2a_, IgG_2b_ and IgG_3_ antibodies in the supernatant of cell cultures were assessed using the LEGENDplex^TM^ Mouse Immunoglobulin Isotyping Panel (BioLegend). For analysis of IgM, probes were diluted 1:5. For all other isotypes, the pure supernatant was applied. Concentrations in pg/mL are depicted as dot plots according to standard curve calculations. Graphs show n=6 mice per genotype from 2 independent experiments as mean ± SEM. Significance was determined by *Student’s t-test*. A p-value of <0.05 was considered statistically significant with “§” indicating significant changes of Wt-cells, “#” of SLy2-Ko-cells and asterisks reflecting significant differences between both genotypes (§ # *p<0.05).

Supplementary Figure 5: Relative IL-9 Expression in BMSCs from SLy2-Ko and Wt littermate Mice.

IL-9 mRNA-expression of isolated BMSCs after 48 hours of cell culture as determined by RT-PCR. The graph depicts the relative expression of IL-9 normalized to ß-Actin and is shown as mean ± SEM. n=6 mice are illustrated from 2 independent experiments.

Supplementary Figure 6: CpG-DNA-induced Proliferation of isolated peritoneal and splenic B-1 Cells.

B-1 cells were isolated from the pool of total splenocytes or peritoneal washouts by the use of magnetic activated cell sorting (MACS) beads (Miltenyi Biotech). Subsequently, cells were stained with the CellTrace^TM^ Violet Proliferation Kit (Invitrogen) according to manufacturers protocol and cultured in
500 µL of complete medium either unstimulated (US) or with 20 µg/mL CpG-DNA (Bio-techne). After
48 hours of stimulation at 37°C and 5% CO_2_, cells were stained with anti-CD19 FITC, anti-CD43 PE-Cy7, anti-CD5 APC and anti-IgM PE and analyzed via flow cytometry. Dot plots show n=3-6 mice per genotype and data are presented as mean ± SEM. Significance was determined by *One-way ANOVA* with multiple comparisons and a p-value of <0.05 was considered statistically significant (**p<0.01 ***p<0.001).
